# Zoledronate Enhances Osteocyte-Mediated Osteoclast Differentiation by IL-6/RANKL Axis

**DOI:** 10.3390/ijms20061467

**Published:** 2019-03-22

**Authors:** Hyung Joon Kim, Ha Jin Kim, YunJeong Choi, Moon-Kyoung Bae, Dae Seok Hwang, Sang-Hun Shin, Jae-Yeol Lee

**Affiliations:** 1 Department of Oral Physiology, BK21 PLUS Project, and Institute of Translational Dental Sciences, School of Dentistry, Pusan National University, Yangsan 50612, Korea; hjoonkim@pusan.ac.kr (H.J.K.); ya120010@naver.com (H.J.K.); celinechoi@pusan.ac.kr (Y.C.); mkbae@pusan.ac.kr (M.-K.B.); 2 Department of Oral and Maxillofacial Surgery, School of Dentistry, Pusan National University, Yangsan 50612, Korea; dshwang@pusan.ac.kr (D.S.H.); ssh8080@pusan.ac.kr (S.-H.S.); 3 Biomedical Research Institute, Pusan National University Hospital, Busan 49241, Korea

**Keywords:** bisphosphonate, osteocyte, osteoclast, RANKL, interleukin-6

## Abstract

Bisphosphonates are one of the most widely used synthetic pyrophosphate analogues for the treatment of bone resorbing diseases such as osteoporosis, multiple myeloma, and bone metastases. Although the therapeutic usefulness of bisphosphonates mainly depends on their anti-osteoclastogenic effect, a severe side-effect of bisphosphonates called bisphosphonate-related osteonecrosis of the jaw (BRONJ) could not be explained by the anti-osteoclastogenic effect of bisphosphonates. In the present study, we have evaluated the changes in osteoclastogenesis- or osteoblastogenesis-supporting activities of osteocytes induced by bisphosphonates. Zoledronate, a nitrogen-containing bisphosphonate, markedly increased both the receptor activator of nuclear factor kB ligand (RANKL) as well as sclerostin in osteocyte-like MLO-Y4 cells, which were functionally revalidated by osteoclast/osteoblast generating activities of the conditioned medium obtained from zoledronate-treated MLO-Y4 cells. Of note, the zoledronate treatment-induced upregulation of the RANKL expression was mediated by autocrine interleukin-6 (IL-6) and subsequent activation of the signal transducer and activator of transcription 3 (STAT3) pathway. These results were evidenced by the blunted RANKL expression in the presence of a Janus activated kinase (JAK2)/STAT3 inhibitor, AG490. Also, the osteoclastogenesis-supporting activity was significantly decreased in zoledronate-treated MLO-Y4 cells in the presence of IL-6 neutralizing IgG compared to that of the control IgG. Thus, our results show previously unanticipated effects of anti-bone resorptive bisphosphonate and suggest a potential clinical importance of osteocytes in BRONJ development.

## 1. Introduction

After the monumental studies by the Fleisch group in the 1980s, bisphosphonates (BPs) have become the most commonly used anti-bone resorptive drug for the treatment of osteoporosis, multiple myeloma, Paget’s disease, and bone-metastatic cancers [1,2]. BPs are synthetic analogues of inorganic pyrophosphate (PPi), possessing a non-hydrolysable nature as well as strong affinity with bone [3,4]. When BPs are repeatedly administered, they preferentially accumulate in the hydroxyapatite of bone then integrate into osteoclasts during the bone resorption process. The cytotoxic effects of BPs are mainly exerted on osteoclasts.

The mechanism of action is different depending on the type of BPs. BPs are classified into nitrogen-containing BPs (N-BPs) and non-nitrogen-containing BPs (non-N-BPs) according to the existence of nitrogen in their side-chain [5]. N-BPs, such as ibandronate, alendronate, and zoledronate inhibit the mevalonate pathway and subsequent prenylation of membrane bound G-proteins which result in overall signal transduction defects in osteoclasts [6,7]. On the other hand, non-N-BPs, such as etidronate, medronate, and clodronate inhibit osteoclasts by producing non-hydrolyzable cytotoxic ATP analogues within osteoclasts [8,9]. Although both BPs bind to the hydroxyapatite on the bone surface, N-BPs are known to have a stronger anti-osteoclastic effect than non-N-BPs [2,4]. 

In spite of its advantageous medical effects, a rare yet severe side effect of BPs, called bisphosphonate-related osteonecrosis of the jaw (BRONJ), was first reported in 2003 [10]. The major symptoms of BRONJ are the exposure of necrotic jaw bone associated with inflamed oral soft tissues. With the rapid transition to an aging society, anti-bone resorptive drugs including BPs are more frequently prescribed and consequently, the incidences of BRONJ are increasing. Although oral infections and surgical dental treatments, such as tooth extractions or implants, are regarded as a triggering event for the development of BRONJ [2,11], the exact molecular pathway of BRONJ pathogenesis remains elusive. In addition, the recently reported observations addressing the occurrences of osteonecrosis of the jaw with anti-angiogenic reagents or anti-resorptive drugs beside denosumab, an anti-receptor activator of nuclear factor kB ligand (RANKL) drug that blocks osteoclast formation, made it more complicated to ravel out the pathogenesis of BRONJ [12]. Therefore, to accommodate the increasing occurrences of non-BPs-mediated osteonecrosis of the maxilla and the mandible, the American Association of Oral and Maxillofacial Surgeons favors to use the term medication-related osteonecrosis of the jaw (MRONJ) instead of BRONJ [12,13]. 

Even though the therapeutic targets of BPs are mostly osteoclasts, increasing evidences suggest unanticipated effects of BPs on other type of cells, which could provide important clues about the pathogenesis of BRONJ. BPs have substantial effects on other cells involved in bone homeostasis, such as osteoblasts [14] and keratinocytes [15]. The bone maintains its homeostasis by undergoing a continual remodeling process. In the bone tissue, the most abundant bone cells are osteocytes. Osteocytes are differentiated cells originating from osteoblasts and they become embedded in the bone matrix. During the last decade, researchers have focused on the role of osteocytes and highlighted their role as a master regulator of bone homeostasis [16,17]. Osteocytes secrete two important factors to orchestrate the bone remodeling: Sclerostin, a Wnt antagonist, which reduces bone formation, and receptor-activator of NF-kB ligand (RANKL), an essential cytokine for osteoclast differentiation [16,17]. There have been studies reporting the cell-protective effects of BPs against estrogen-depletion-mediated apoptosis of osteocytes [18]. However, the role of BPs on the osteoclastogenesis supporting activity of an osteocyte has not been clearly described yet. 

In this study, we have found that the increased expression of RANKL in MLO-Y4 cells treated with zoledronate significantly stimulated the differentiation of osteoclast precursor cells in a co-culture environment. Furthermore, zoledronate-induced osteocytic expression of RANKL was blunted by AG490, a Janus activated kinase (JAK2) inhibitor, and interleukin-6 (IL-6) neutralizing antibody, which suggests the involvement of an IL-6/JAK2/signal transducer and activator of transcription 3 (STAT3) pathway in the zoledronate-induced RANKL expression. Taken together, our results showed a novel role of BPs beyond the anti-osteoclast effect and might lead us a step closer to understanding the pathogenesis of BRONJ.

## 2. Results

### 2.1. Zoledronate, a Nitrogen-Containing Bisphosphonate, Significantly Increased RANKL and Sclerostin Expression from Osteocyte-Like MLO-Y4 Cells

**Figure 1:**
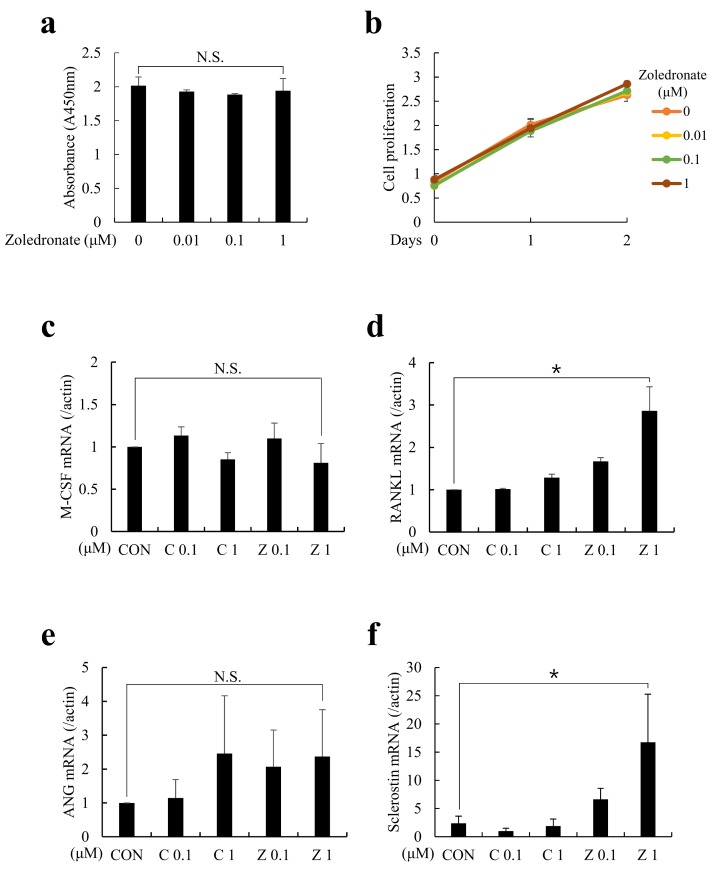
Zoledronate increased receptor activator of nuclear factor kB ligand (RANKL) and sclerostin mRNA expressions in MLO-Y4 cells. MLO-Y4 cells were cultured in the presence of indicated doses of clodronate (C, 0.1 or 1 µM) or zoledronate (Z, 0.1 or 1 µM) for 48 h. (**a**,**b**) Effects of increasing concentrations of zoledronate on MLO-Y4 cells. Cell Counting Kit-8 (CCK-8) assay showed that zoledronate did not reduce cell viability at all concentrations. (**c**–**f**) At the end of culture, total RNAs were isolated and the mRNA expressions of (**c**) Macrophage-colony stimulating factor (M-CSF), (**d**) RANKL, (**e**) angiogenin (ANG), and (**f**) sclerostin were examined by quantitative real-time PCR analyses. CON refers to the control vehicle, phosphate-buffered saline (PBS). The quantitative data are presented as mean ± SD (* *p* < 0.05).

To evaluate the direct effects of BPs on the osteocytic cytokine expressions, MLO-Y4 cells, a well-established osteocyte-like cell line, were cultured in the presence of clodronate (non-N-BPs) or zoledronate (N-BPs) for 48 h. Before testing for the expressions of various cytokines, the cytotoxicity of zoledronate on MLO-Y4 cells was measured. Compared with the control, cell proliferation was not significantly decreased by increasing concentrations of zoledronate. Next, macrophage colony-stimulating factor (M-CSF) and RANKL were examined to assess the effect of BPs on osteoclastogenesis. Also, angiogenin (ANG) and sclerostin were examined to assess the effect of BPs on angiogenesis and osteoblastogenesis, respectively. As shown in Figure 1a,c, the mRNA expressions of M-CSF and ANG were unchanged in the presence of both kinds of BPs. However, RANKL and sclerostin mRNAs were markedly increased in zoledronate-treated MLO-Y4 cells (Figure 1b,d). Notably, the addition of zoledronate, but not that of clodronate, triggered dose-dependent elevation of RANKL and sclerostin expressions.

### 2.2. Conditioned Medium (C.M.) Obtained from Zoledronate-Treated MLO-Y4 Cells Reduced by Osteoblast Differentiation

**Figure 2:**
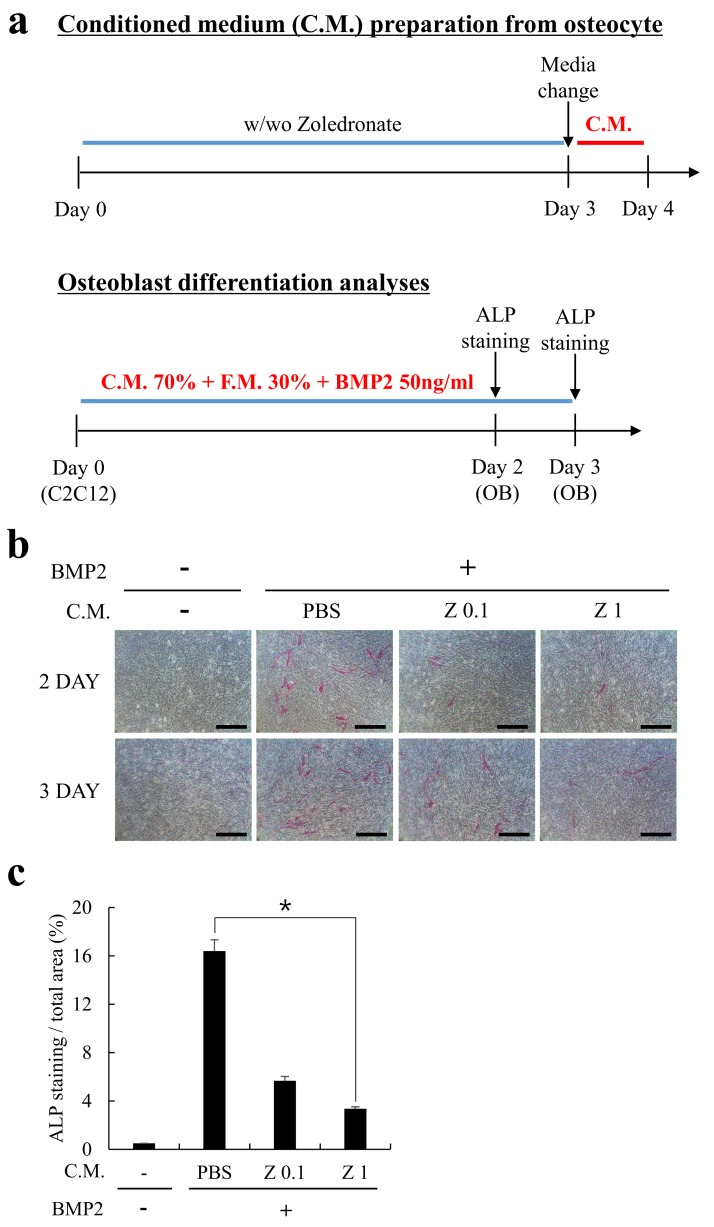
Conditioned medium (C.M.) from zoledronate-treated MLO-Y4 cells functionally reduced the osteoblastic differentiation of C2C12 cells. (**a**) A schematic diagram of C.M. preparation and osteoblastogenesis activity analyses. MLO-Y4 cells were cultured in the absence or presence of zoledronate (0.1 or 1 µM) for 3 days, then the cultured medium was exchanged with fresh medium (without zoledronate) and the C.M. was collected for one more day. Separately, C2C12 cells were allowed to differentiate into osteoblasts in the presence of a mixed osteogenic medium (C.M. 70% + F.M. 30% + BMP2 50 ng/mL) for 2 or 3 days. F.M. stands for fresh medium; (**b**) C2C12 cells were cultured as in (**a**) and the osteogenic differentiation was assessed by alkaline phosphatase (ALP) staining on days 2 and 3. Bars, 200 µm; (**c**) ALP stained images were quantified from experiments in panel (**b**). The quantitative data are presented as mean ± SD (* *p* < 0.05).

Sclerostin is a glycoprotein secreted by osteocytes which inhibits osteoblast activity by antagonizing Wingless (Wnt) signaling and bone morphogenetic (BMP) signaling [17,19]. Because zoledronate significantly induced the expression of sclerostin from MLO-Y4 cells (Figure 1d), we next examined the functional effect of the conditioned medium (C.M.) from zoledronate-treated MLO-Y4 cells. MLO-Y4 cells were cultured with or without zoledronate (0.1 or 1 µM) as shown in Figure 2a, and the C.M. was harvested and mixed into the osteogenic culture medium of C2C12 cells, a mouse myoblast cell line. As expected, the administration of C.M. harvested from zoledronate-treated MLO-Y4 cells distinctly diminished the osteoblastic differentiation of C2C12 cells (Figure 2b,c). This result suggests that zoledronate-induced upregulation of sclerostin expression is functional.

### 2.3. Zoledronate Treatment Increased the Expression of Osteoclastogenesis Supporting Factor from MLO-Y4 Cells

**Figure 3:**
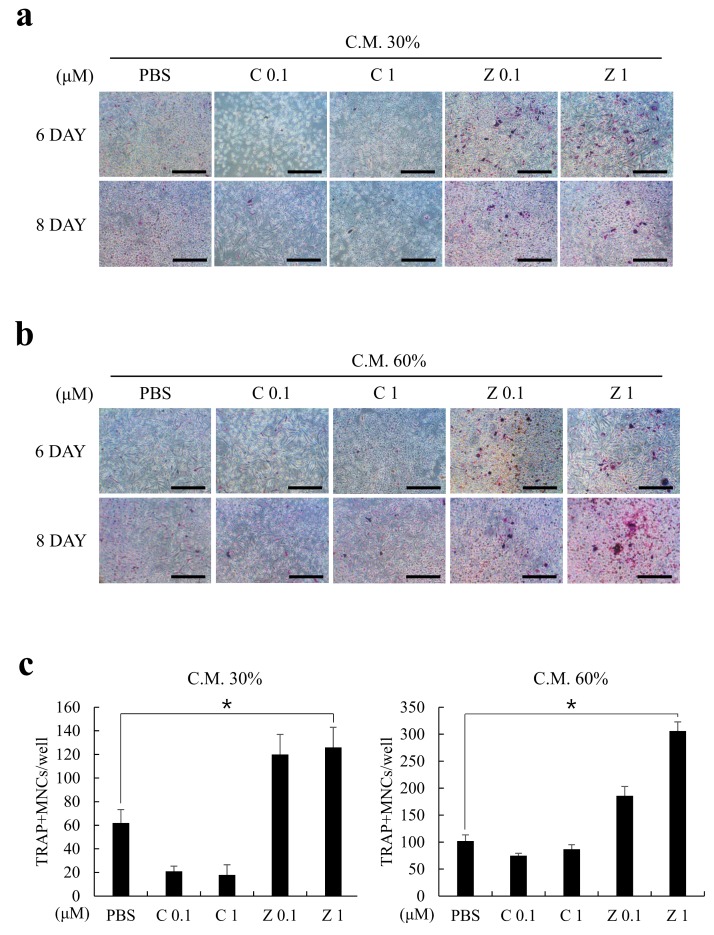
Zoledronate, but not clodronate, induced the secretion of pro-osteoclastogenic factors from MLO-Y4 cells. MLO-Y4 cells were cultured with or without clodronate (C; 0.1 or 1 µM) or zoledronate (Z; 0.1 or 1 µM) and the C.M. was collected as in Figure 2a (upper). (**a**) Osteoclast differentiation was induced on bone marrow-derived macrophages (BMMs) by the medium containing C.M. 30% + F.M. 70% for the indicated days (without extrinsic addition of M-CSF & RANKL). Osteoclasts were visualized by tartrate-resistant acid phosphatase (TRAP) staining and the representative images are shown. Bars, 200 µm; (**b**) BMMs were cultured as in (**a**) with C.M. 60% + F.M. 40% mixed medium; (**c**) TRAP+ multinucleated cells (MNCs) were quantified from (**a**,**b**) experiments. All quantitative data are presented as mean ± SD (* *p* < 0.05).

M-CSF and RANKL are well-established, essential osteoclastogenic cytokines [20,21]. Because the MLO-Y4 cells constantly expressed M-CSF regardless of BPs treatment (Figure 1a) and the expression of RANKL was significantly upregulated by zoledronate rather than clodronate (Figure 1b), we hypothesized that the C.M. obtained from zoledronate-treated MLO-Y4 cells would induce greater osteoclast formation than clodronate-treated cells. To test this hypothesis, clodronate- or zoledronate-treated C.M. was prepared from MLO-Y4 cells as in Figure 2a, and the osteoclastogenesis-supporting activities were analyzed using a bone marrow-derived macrophages (BMMs)-based osteoclast formation model. In accordance with Figure 1 results, the C.M. alone sufficiently induced osteoclast differentiation, even though the C.M. did not contain additional M-CSF or RANKL (Figure 3a,b). Importantly, the C.M. from zoledronate-treated MLO-Y4 cells resulted in a greater osteoclast formation in comparison with the clodronate-treated group. Furthermore, the osteoclast formation was proportional to the percentage of mixed C.M. (Figure 3c). Again, these results indicated that zoledronate, but not clodronate, induced pro-osteoclastogenic cytokine expression, probably RANKL, from osteocytes.

### 2.4. The Autocrine Expression of IL-6 Upon Zoledronate Treatment Resulted in RANKL Expression in MLO-Y4 Cells through JAK2/STAT3 Pathway

**Figure 4:**
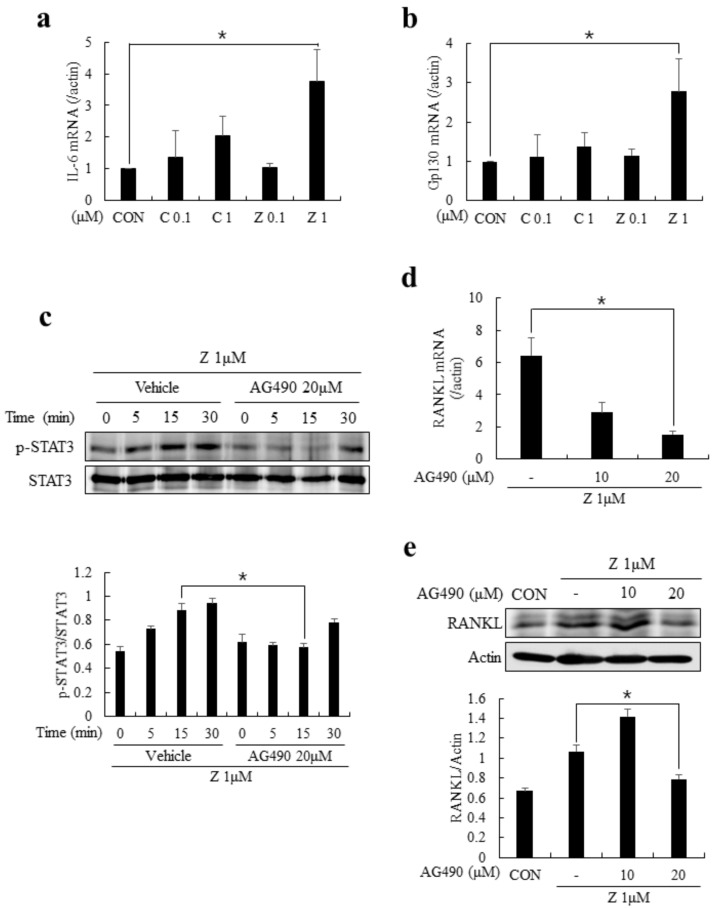
Zoledronate enhanced the RANKL expression via interleukin-6 (IL-6)/Janus activated kinase (JAK2)/ signal transducer and activator of transcription 3 (STAT3) pathway in MLO-Y4 cells. (**a**,**b**) MLO-Y4 cells were cultured in the presence of indicated doses of clodronate (C; 0.1 or 1 µM) or zoledronate (Z; 0.1 or 1 µM) for 2 days and the mRNA expressions of IL-6 and gp130 were examined by quantitative real-time PCR; (**c**) MLO-Y4 cells were cultured in the medium containing zoledronate (1 µM) for 2 days and serum-starved for 5 h. The cells were pretreated with vehicle (DMSO) or AG490 (20 µM) for another 1 h and re-stimulated with zoledronate. The phosphorylation of STAT3 was evaluated by immunoblotting (upper). The relative band intensity of p-STAT3 was normalized by STAT3 with densitometry and the quantitative results are shown as graphs (lower); (**d**,**e**) MLO-Y4 cells were cultured in the presence of zoledronate (1 µM) together with AG490 (0, 10, and 20 µM) for 2 days. After the culture, the expression of RANKL was determined by quantitative real-time PCR (**d**) or immunoblotting (**e**). Quantitative data are presented as mean ± SD (* *p* < 0.05).

Previous studies evidenced that the fine-tuned regulation of bone remodeling by osteoblasts, osteoclasts, and osteocytes required inflammatory cytokines [21,22]. Among these inflammatory cytokines, IL-6 has been known to be a potent stimulator of RANKL expression in osteoblasts and osteocytes [23,24]. The association of IL-6/IL-6 receptor and glycoprotein130 (gp130) could activate Janus activated kinase (JAK) and lead to the phosphorylation of signal transducer and activator of transcription 3 (STAT3), facilitating the RANKL expression [23]. Therefore, finding that zoledronate can induce the upregulation of RANKL led us to hypothesize that IL-6 may be controlling the RANKL expression in MLO-Y4 cells in the presence of zoledronate. As shown in Figure 4a,b, the mRNA expressions of IL-6 and gp130 were significantly increased in the presence of zoledronate (at 1 µM). In addition, the phosphorylation of STAT3 was substantially induced after zoledronate stimulation, whereas it was markedly reduced by treating AG490, a JAK2 inhibitor (Figure 4c). Of note, the zoledronate-induced upregulation of RANKL expression was strongly inhibited by AG490 at both mRNA and protein levels (Figure 4d,e). These results suggest that the zoledronate-mediated regulation of IL-6 production and serial activation of JAK2/STAT3 are crucial for the RANKL expression from MLO-Y4 cells.

### 2.5. AG490 and IL-6 Neutralizing Antibody Inhibited the Expression of RANKL and Reduced the Differentiation of Osteoclasts co-Cultured with MLO-Y4 Cells

**Figure 5:**
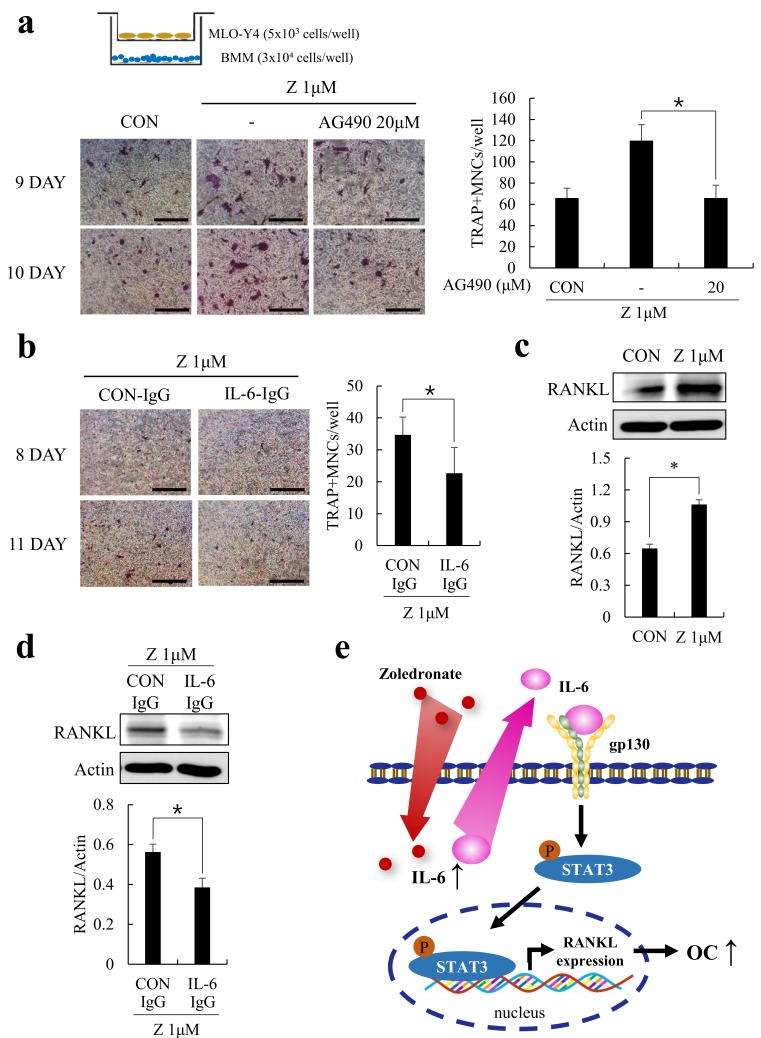
Zoledronate-induced upregulation of RANKL was reduced by AG490 or IL-6 neutralization. (**a**) MLO-Y4 cells were pretreated with the indicated combinations of zoledronate (1 µM) or AG490 (20 µM) for 2 days. The pretreated MLO-Y4 cells were seeded on the upper chamber, and BMMs were seeded on the lower chamber then allowed to differentiate into osteoclasts in the zoledronate/AG490-free condition. At the end of culture, osteoclasts were stained for TRAP activity and the number of TRAP+ MNCs were quantified; (**b**) MLO-Y4 cells were pretreated and co-cultured with BMMs as in (a) in the presence of a control antibody (CON-IgG) or IL-6 neutralizing antibody (IL-6-IgG). Representative TRAP stained images and quantitative TRAP+ MNCs are shown. Bars, 200 µm; (**c**) MLO-Y4 cells were cultured with or without zoledronate (1 µM) for 2 days and the protein expression of RANKL was examined by immunoblotting; (**d**) MLO-Y4 cells were treated with zoledronate (1 µM) together with a control antibody (CON-IgG) or IL-6 neutralizing antibody (IL-6-IgG) for 2 days. RANKL protein expression was evaluated by immunoblotting. All quantitative data are presented as mean ± SD (* *p* < 0.05) (**e**) schematic diagram for the effect of zoledronate on the osteoclastogenesis supporting activity of MLO-Y4 cells. In the presence of zoledronate, increased expression of IL-6 triggers the elevated RANKL expression via JAK2/STAT3 pathway, which potentiates the osteoclast formation.

Since AG490, a JAK2 inhibitor, reduced the mRNA and protein expressions of RANKL in MLO-Y4 cells, we next sought to investigate its effect on the osteoclastogenesis-supporting activity of MLO-Y4 cells using the BMMs-MLO-Y4 co-culture system. By employing the trans-well system, the exchange of soluble factors such as RANKL was possible without the direct contact of two cell types. Also, to exclude the anti-osteoclastogenic effect of zoledronate, MLO-Y4 cells were pretreated with zoledronate and co-cultured with BMMs in zoledronate-free conditions. As shown in Figure 5a, zoledronate-treated MLO-Y4 cells significantly enhanced osteoclast formation compared to the non-treated MLO-Y4 cells, but this effect was reversed by AG490. To further elucidate the potential contribution of IL-6 in RANKL expression, we examined the effect of IL-6 neutralizing antibody (IL-6-IgG) within the co-culture system. In accordance with our previous results, the IL-6-IgG treated MLO-Y4 cells showed reduced osteoclastogenic activity (Figure 5b). In these experiments, zoledronate still induced RANKL expression (Figure 5c), but it was substantially reduced in the presence of IL-6-IgG (Figure 5d). These data support the role of zoledronate in IL-6 mediated RANKL expression which required JAK2/STAT3 activation (Figure 5e).

## 3. Discussion

BPs have been prescribed to various diseases involving bone-related abnormalities, such as Paget’s disease, osteoporosis, and bone metastases [4,25]. Although the most anticipated effects of BPs might be an inhibitory action on osteoclasts, and increasing evidence reports that other cell types could be affected by BPs. Most importantly, BPs are known to modulate the proliferation and differentiation of osteoblasts [25,26]. Several studies have observed the enhancing effects of BPs on osteoblast proliferation and mineralization, but other studies have shown the reducing effects on osteoblast activity [27,28,29,30]. Although the results are varying, or even conflicting, depending on the type of BPs or the cell sources used, BPs’ control of the osteoblastic lineage cells is a certainty. Moreover, the modulation of osteoblast metabolism by BPs underlines the importance of BPs in the regulation of bone homeostasis in an indirect manner, because the fine-tuned bone remodeling is mediated by the coupled communication between the osteoblasts and osteoclasts. In addition, this notion also suggests a plausible influence of BPs on another type of cell, osteocytes, which are matured cells of osteoblastic lineage.

Osteocytes, the most abundant cells in the bone tissue, are bone-matrix embedded, terminally differentiated cells of osteoblasts. At first, osteocytes were considered as inactive, quiescent cells merely residing in the mineralized tissue, but nowadays, they are becoming accepted as a master regulator of bone remodeling adapting to mechanical forces or hormonal changes [16,17]. In 2002, Zhao et al. demonstrated that MLO-Y4 osteocyte-like cells can stimulate and support osteoclast formation without the introduction of exogenous osteotropic factors in vitro [31]. Thereafter, researches using genetically modified mice have shown that gene deletion of RANKL from osteocytes resulted in a systemic osteopetrosis due to defects in osteoclast formation, suggesting that osteocytes are an essential source of RANKL [32,33]. More recently, IL-6 has been shown to stimulate RANKL expression in osteocytes via the JAK2/STAT3 pathway [23]. Among many inflammatory cytokines, IL-6 was already known to induce RANKL production from osteoblasts via the activation of STAT3 [34]. Therefore, it seems like the production of RANKL is induced by a common pathway, at least in osteoblasts and osteocytes. In accordance with previous reports, we observed the increase in IL-6 expression and subsequent upregulation of RANKL expression. BPs, specifically zoledronate, induced RANKL expression in a IL-6 dependent manner, because the expression of RANKL in MLO-Y4 cells was impeded by a IL-6 neutralizing antibody (Figure 5c,d). Besides IL-6, zoledronate treatment also induced the upregulation of sclerostin in MLO-Y4 cells (Figure 1d). Sclerostin is a major product of osteocytes, and the mutation or deletion of the sclerostin gene, SOST gene, causes high bone mass disease in humans and mice [35,36]. This suggests an important role of sclerostin in bone mass regulation. Although the inhibitory action of sclerostin on the initiation of BMP and Wnt signaling is largely known [17,35], the detailed signaling pathways of sclerostin is still elusive. Until recently, the focus of research on sclerostin has been centered on its anti-osteoblastogenic action because of its known roles in BMP/Wnt signaling; however, several evidences indicate the direct action of sclerostin on bone resorption and osteoclastogenesis. Notably, sclerostin has been shown to upregulate the expression of RANKL and downregulate that of osteoprotegerin (OPG) in pre-osteocytes and MLO-Y4 cells [37]. In our experiment, zoledronate simultaneously increased the sclerostin mRNA expression as well as that of RANKL in MLO-Y4 cells (Figure 1b,d), which could lead us to speculate sclerostin as a causative factor for the upregulation of RANKL. However, it is highly unlikely because low doses of zoledronate (0.1 µM) had a minimal effect on sclerostin induction (Figure 1d), but a strong pro-osteoclastogenic activity was observed by the same dose of zoledronate (Figure 3c). Nevertheless, the role of sclerostin in association with BP-mediated alterations on the osteoclast supporting activity of osteocytes should be examined in further studies. 

In summary, the present study showed that zoledronate enhanced osteocyte-mediated osteoclastogenesis through elevated expression of IL-6 and subsequent RANKL expression. Interestingly, this phenomenon was not observed by non-N-BPs, clodronate in particular, and JAK2/STAT3 pathways seemed to be involved in zoledronate-induced RANKL expression in MLO-Y4 cells. These results suggest a previously unanticipated side effect of BPs, which are commonly used to inhibit osteoclast activity. Whether these results can be transferred to unveiling the mechanism leading to the pathogenesis of BRONJ or not is questionable. Nevertheless, our findings would contribute to expanding the current understanding of bone metabolism and novel actions of BPs.

## 4. Materials and Methods

### 4.1. Reagents

Clodronate was purchased from Cayman (E. Ellsworth, MI, USA) and dissolved in phosphate buffer saline (PBS). Zoledronate was purchased from Sigma-Aldrich (St. Louis, MO, USA) and dissolved in phosphate buffer saline (PBS). AG490 was purchased from Calbiochem (La Jolla, CA, USA) and dissolved in dimethyl sulfoxide (DMSO). Control-IgG (Rat IgG2a Control) and IL-6-IgG (Anti-mIL-6-IgG) were purchased from InvivoGen (San Diego, CA, USA). Antibodies against phosphor-STAT3 and STAT3 were obtained from Cell Signaling Technology (Danvers, MA, USA). Anti-RANKL antibody was purchased from Santa Cruz Biotechnology, Inc. (Santa Cruz, CA, USA). The Leukocyte Acid Phosphatase (TRAP) kit, Leukocyte Alkaline Phosphatase (ALP) kit, and anti-β-actin antibody were obtained from Sigma-Aldrich (St. Louis, MO, USA).

### 4.2. Cell Cultures

The murine osteocyte-like cell line MLO-Y4 (Kerafast, MA, Boston) was plated on type I collagen (Santa Cruz, CA, USA) coated dishes and cultured in a MEM medium supplemented with 5% fetal bovine serum (FBS) and 2.5% penicillin at 37 °C in a humidified 5% CO_2_ atmosphere.

C2C12 myoblast cell line was obtained from American Type Culture Collection (Manassas, VA, USA) and grown with DMEM/high glucose supplemented with 10% FBS in 5% CO_2_ at 37 °C. The osteoblast differentiation was induced with 50 ng/mL of BMP2 (R&D Systems Inc., Minneapolis, MN, USA) in the presence of indicated conditioned medium (C.M.) for up to 3 days and the cells were stained for ALP to assess osteoblast differentiation.

BMMs (bone marrow-derived macrophages) were isolated from ICR mice and used as precursor cells for osteoclasts as previously described [38]. The osteoclast differentiation was induced with C.M. obtained from MLO-Y4 cells under indicated experimental conditions. No exogenous osteoclastogenic factors such as M-CSF and RANKL were added to this culture. Although the exact time required for the osteoclast formation varied slightly between experiments, the C.M. induced osteoclastogenesis was generally slower than that of M-CSF and RANKL. At the end of the culture, cells were stained for TRAP activity and TRAP+ multinucleated cells (MNCs) having three or more nuclei were counted as osteoclast.

### 4.3. Cytotoxicity Assay

Cytotoxicity of zoledronate was evaluated using the Cell Counting Kit-8 (CCK-8; Dojindo Laboratories, Kumamoto, Japan). MLO-Y4 cells were plated in 48-well plates at a density of 1.5 × 10^5^ cells/well in triplicate and treated with increasing concentrations of zoledronate. After a 16 h incubation, 20 µL of the CCK-8 solution was added to each well and the plate was incubated for an additional 3 h. The absorbance of each well was measured at 450 nm with a reference at 655 nm using the Benchmark microplate reader (Bio-Rad Laboratories, Hercules, CA, USA).

### 4.4. Conditioned Medium (C.M.) Preparation from MLO-Y4 Cells

MLO-Y4 cells (2 × 10^5^) were seeded on 6-well culture dishes with 2 mL of complete α-MEM medium (10% FBS plus 1% antibiotics) in the presence or absence of clodronate/zoledronate (0.1 or 1 µM) for 3 days. On day 3, the culture medium was replaced by fresh α-MEM to remove any remaining BPs and the conditioned medium was harvested the next day. The collected C.M. was immediately centrifuged to eliminate cell debris and filtrated with syringe filter (0.2 µm pore size) before use.

### 4.5. Co-Culture of MLO-Y4 Cells and BMMs

To examine the osteoclastogenesis supporting activity of MLO-Y4 cells, MLO-Y4 cells and BMMs were co-cultured in the trans-well system with 8 µm pore filters (Corning, NY, USA). At first, MLO-Y4 cells (5 × 10^3^) were seeded on the upper chamber of the trans-well plate with or without the indicated combinations of zoledronate, AG490, CON-IgG, and IL-6-IgG for 2 days (Figure 5). After 2 days of pretreatment, BMMs (3 × 10^4^) were seeded on the lower chamber in complete α-MEM medium and allowed to differentiate into osteoclasts in a MLO-Y4 cell-dependent manner. The shared culture medium was changed with α-MEM every 2 days. After the culture, the cells on the lower chamber were stained for TRAP activity.

### 4.6. Quantitative Real-Time Polymerase-Chain Reaction (PCR) Analyses

For real-time PCR analysis, 2 μg of cDNAs were amplified with SYBR green PCR master mix (Applied Biosystems) in a MicroAmp optical tube (Applied Biosystems) for 40 cycles of denaturation (15 sec) at 95 °C and amplification (60 sec) at 60 °C in AB7500 instruments (Applied Biosystems). The primer sets used in PCR were as follows: M-CSF, 5′-TTC TCA GAG GAC AGA GGG CA-3′ (forward) and 5′-GTC TGT CCC CAT GGT TTG GT-3′ (reverse); RANKL, 5′-AGG CTG GGC CAA GAT CTC TA-3′ (forward) and 5′-GTC TGT AGG TAC GCT TCC CG-3′ (reverse); ANG, 5′-ACT TCA GTC CTA CCC TGA GCA-3′ (forward) and 5′-CTG AGC ATG CCT GGG TCA AA-3′ (reverse); Sclerostin, 5′-CAG GAA TGA TGC CAC AGA GGT-3′ (forward) and 5′-GCA GCT GTA CTC GGA CAC AT-3′ (reverse); IL-6, 5′-TTT CCT CTG GTC TTC TGG AGT-3′ (forward) and 5′-TGT GAC TCC AGC TTA TCT CTT GG-3′ (reverse); IL-6R(gp130), 5′-CCG CGT ACA CAG ATG AAG GT-3′ (forward) and 5′-CTA AGC ACA CAG GCA CGA CT-3′ (reverse); Actin, 5′-TCT GGC ACC ACA CCT TCT AC-3′ (forward) and 5′-TAC GAC CAG AGG CAT ACA GG-3′ (reverse).

### 4.7. Western Blotting

Western blotting (immunoblotting) was conducted following a standard experimental procedure. In brief, the cultured MLO-Y4 cells were disrupted using a lysis buffer (50 mM Tris; pH 8.0, 150 mM NaCl, 0.5% sodium deoxycholate, 1mM EGTA, 1% Triton X-100, 10 mM NaF, and complete protease inhibitor cocktail). The equal concentrations of cell lysates (30–45 µg) were divided into the same amounts for multiple western blotting, and the lysates were resolved by 8%–10% SDS-polyacrylamide gel electrophoresis. Separated protein bands were transferred onto nitrocellulose membranes and followed by blocking with 5% skim milk for 1 h. The expression of respective protein was determined by immunoreactivity of membranes with chemiluminescence reagents. Among the multiple results, only the representative blots were shown and the band images were quantified using ImageJ software (version 2, NIH, MD, USA).

### 4.8. Statistics

All of the data presented in this study are representative results of at least three independent experiments performed in triplicate unless otherwise mentioned. Statistical significance levels were calculated with the Wilcoxon–Mann–Whitney U Test [39] using IBM SPSS Statistics 25 (Armonk, NY, USA). Differences with *p* < 0.05 or over were regarded as significant (* *p* < 0.05).

## Abbreviations

RANKL	Receptor Activator of Nuclear Factor kB Ligand
BRONJ	Bisphosphonate-Related Osteonecrosis of the Jaw
BMM	Bone Marrow-derived Macrophage
M-CSF	Macrophage-Colony Stimulating Factor
